# Cadmium-Containing Carbonic Anhydrase CDCA1 in Marine Diatom *Thalassiosira weissflogii*

**DOI:** 10.3390/md13041688

**Published:** 2015-03-25

**Authors:** Vincenzo Alterio, Emma Langella, Giuseppina De Simone, Simona Maria Monti

**Affiliations:** Institute of Biostructures and Bioimaging—National Research Council (CNR), Via Mezzocannone 16, I-80134 Naples, Italy; E-Mails: vincenzo.alterio@cnr.it (V.A.); emma.langella@cnr.it (E.L.); gdesimon@unina.it (G.D.S.)

**Keywords:** diatom, *Thalassiosira weissflogii*, carbonic anhydrase, cadmium, zinc

## Abstract

The Carbon Concentration Mechanism (CCM) allows phytoplakton species to accumulate the dissolved inorganic carbon (DIC) necessary for an efficient photosynthesis even under carbon dioxide limitation. In this mechanism of primary importance for diatoms, a key role is played by carbonic anhydrase (CA) enzymes which catalyze the reversible hydration of CO_2_, thus taking part in the acquisition of inorganic carbon for photosynthesis. A novel CA, named CDCA1, has been recently discovered in the marine diatom *Thalassiosira weissflogii*. CDCA1 is a cambialistic enzyme since it naturally uses Cd^2+^ as catalytic metal ion, but if necessary can spontaneously exchange Cd^2+^ to Zn^2+^. Here, the biochemical and structural features of CDCA1 enzyme will be presented together with its putative biotechnological applications for the detection of metal ions in seawaters.

## 1. Introduction

In oceans, concentration of trace metals has a vertical profile, supporting the evidence that these metals are cycled as other nutrients such as phosphate, nitrate and silicate [1]. Phytoplankton takes up elements at the surface contributing to its impoverishment, whereas regeneration occurs at depth by decomposition of sinking organic matter and remineralization [1,2,3]. Like Zn^2+^, also Cd^2+^ follows this type of nutrient-like profile indicating that it participates to the biological uptake by marine phytoplankton, of which diatoms are one of the most representative types [1,3]. Diatoms are responsible for 40% of the net primary production [3,4] and use carbonic anhydrases (CAs) for the acquisition of dissolved inorganic carbon (DIC) by a carbon concentration mechanism (CCM). In this way, also under carbon dioxide limitation, an efficient photosynthesis is guaranteed. CAs (EC 4.2.1.1) are metalloenzymes that catalyze the reversible physiological reaction of the CO_2_ hydration to bicarbonate ion and proton:

CO_2_ + H_2_O ↔ HCO_3_^−^ + H^+^

These ubiquitous enzymes have been found in eukaryotes and prokariotes and were initially grouped into three CA classes: the α-, β- and γ-class [3,5,6,7,8]. The α-CAs are present in vertebrates, bacteria, algae and cytoplasm of green plants; the β-CAs, are mainly localized in bacteria, algae and chloroplasts of monodicotyledons and dicotyledons; the γ-CAs are mainly found in archaea and some bacteria. Two new CAs, namely TWCA1 and CDCA1, were subsequently discovered in marine diatom *T. weissflogii*. These two enzymes showed peculiar molecular and structural features and did not present conserved residues with respect to the other known CAs, thus leading to the definition of two new CA classes, δ and ζ, respectively.

In this review we will focus on the main biochemical, kinetic and structural features of CDCA1. In addition, some biotechnological applications of this enzyme as potential biosensor in marine environment or bioreactor will be discussed. For a detailed review on δ-CAs the reader can instead refer to reference [9].

Currently, CDCA1 is the only member of the ζCA class completely characterized from a biochemical and structural point of view; CDCA1 homolog genes have been identified only in diatom species and the corresponding translated amino acid sequences were clustered into three groups (the Tw group, the Np group, and the Tp group) by phylogenetic analysis [10]. Interestingly, CDCA1 enzyme naturally uses Cd^2+^ to achieve its biological function, providing the first evidence of the biological employment of this metal ion. This finding is of particular interest considering that cadmium is a heavy metal which is usually assumed as a detrimental element associated to toxicity, since it can cause numerous alterations in cell functioning [11]. Indeed, its exposure leads to several pathologies in living organisms [11], such as oxidative stress, lipid peroxidation, alterations in ion homeostasis, DNA damage, initiation of apoptotic and necrotic processes [12,13,14,15,16,17,18,19]. The deleterious effects of this metal are likely due to its capacity to mimic crucial ions thus affecting cellular signaling pathways [11]. The case of CDCA1 represents the unique example of a biologically advantageous Cd-employment, as a consequence of the diatom capability to adapt in response to the low metal environment of the oceans.

## 2. Kinetic and Inhibition Assays

**Figure 1:**
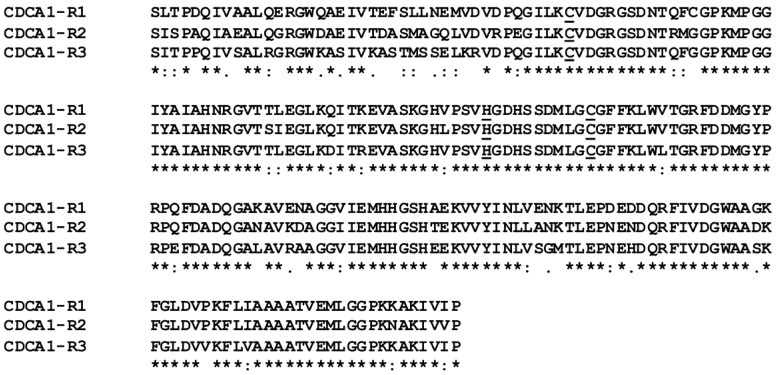
Multiple sequence alignment of CDCA1 single repeats. Conserved residues are indicated with an asterisk (*), while (:) and (.) indicate conservative and semi-conservative substitutions, respectively. Residues involved in metal ion coordination are reported as underlined letters. The alignment was generated with ClustalW, version 1.83.

CDCA1 is a protein of 617 amino acids consisting of three repeats (R1, R2 and R3), which have their own active sites and show high sequence identity with each other (R1/R2 = 82%, R2/R3 = 80%, and R1/R3 = 82%) (Figure 1) [20,21,22].

Despite CDCA1 was originally isolated as a cadmium containing enzyme, it was shown to be a cambialistic protein which can switch from Cd^2+^ to Zn^2+^ and vice versa to perform the catalytic reaction, according to the vanishing concentration of these metal ions in oceans. The facile metal exchange allows the diatom *T. weissflogii* to support the catalytic needs of fast growing diatoms when Zn^2+^ is not sufficient, even though the Cd-bound form of the enzyme is slightly less efficient [3]. The catalytic activity, measured as the rate of interconversion of CO_2_/HCO_3_^−^ by ^18^O exchange [3,23] and stopped-flow assay [22], is greater at higher pH, even greater than that of hCA II which is one of the most catalytically efficient CAs [22]. In fact, Kcat/Km of the Zn-bound full-length CDCA1 ranged from 3.2 × 10^7^ to 8.6 × 10^8^ M^−1^·s^−1^ between pH 6.5 and 9.5, while that of the Cd-CDCA1 protein in the same range of pH ranged from 2.0 × 10^6^ to 1.5 × 10^8^ M^−1^·s^−1^ [3,22]. It is worth noting that a greater catalytic efficiency at higher pHs has also been reported for other investigated CAs [3].

Both the Zn- and Cd-containing R1 fragments were shown to be sensitive to the standard CA inhibitor acetazolamide (5-acetamido-1,3,4-thiadiazole-2-sulfonamide), which inhibited these two enzymes with K_I_s in the range of 58–92 nM. Also other sulfonamide and anion inhibitors were tested showing inhibition constants from the nanomolar to millimolar range [22]. Zn-containing enzyme evidenced a higher affinity for sulfonamide/sulfamate inhibitors compared to the Cd-containing enzyme, likely due to an intrinsic different affinity of such molecules for the two metal ions [24,25].

## 3. Structural Characterization of CDCA1

The three single repeats (R1, R2 and R3) have been fully characterized from a structural point of view using X-ray crystallography [3,24], whereas the full-length protein was recently modelled by using a docking approach [24].

**Figure 2:**
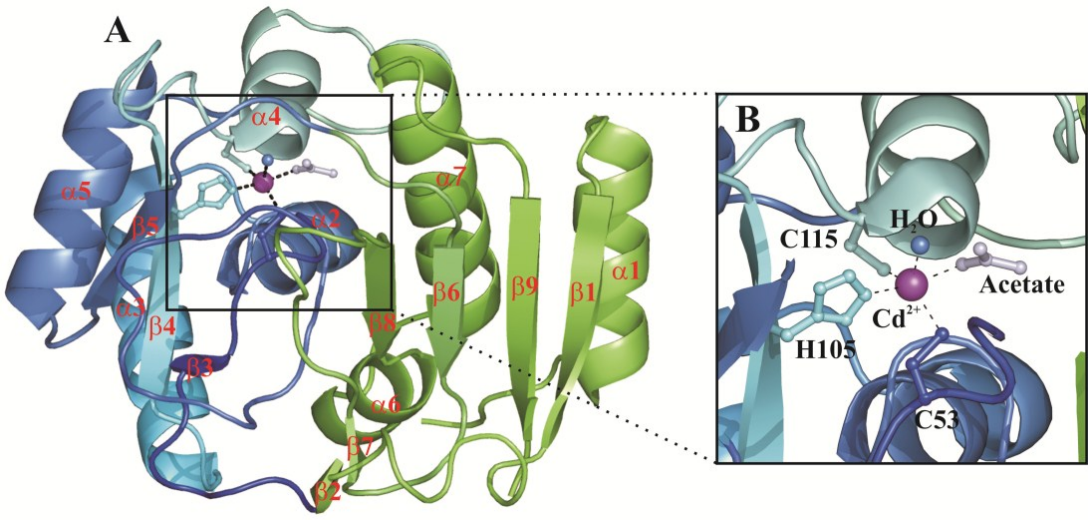
(**A**) CDCA1-R1 overall fold. β-strands and α-helices are shown in cartoon representation and named as reported by [3]. Cd^2+^ is also depicted as purple sphere. The CDCA1 two-lobe architecture is highlighted by different colors: lobe 1 in blue and lobe 2 in green; (**B**) Enlarged view of the CDCA1-R1 active site. Cd^2+^ coordination sphere is shown.

The crystallographic studies revealed that the three repeats, as a consequence of their very high sequence identity, have a very similar 3D structure (r.m.s.d. calculated for the superimposition of the corresponding Cα atoms ranges from 0.4 to 0.5 Å) [24]. Interestingly, they show a completely new fold with respect to the other CA classes characterized so far [3,24], with a structure consisting of seven α-helices, three 3_10_-helices, and nine β-strands organized in three β-sheets (Figure 2A).

The active site is located in a funnel-shaped pocket on the protein surface with the metal ion placed at its bottom where it is coordinated in a highly distorted tetrahedral geometry by three conserved protein residues, *i.e.*, two cysteines and one histidine residue, and a water molecule [3]. In the acetate-bound form, instead, the metal ion shows a trigonal bipyramidal coordination geometry with both the acetate ion and a water molecule coordinated to Cd^2+^ [3,24] (Figure 2B).

It has been speculated by Morel and coworkers that a single CDCA1 repeat is a structural (and functional) mimic of a β-CA dimer [3]. Indeed, each CDCA1 repeat shows an ideal two-lobe architecture, one lobe is made of a three-stranded β-sheet and helices α2-α5 and the other is composed of a four-stranded β-sheet and helices α1, α6 and α7 (Figure 2A). The two lobes align well with two adjacent β-CA monomers and are both necessary for the catalytic activity, as in the case of the β-CA monomers. In fact, the three-stranded lobe contains the three metal-coordinating residues and the four-stranded one encompasses most of the substrate-binding residues.

**Figure 3:**
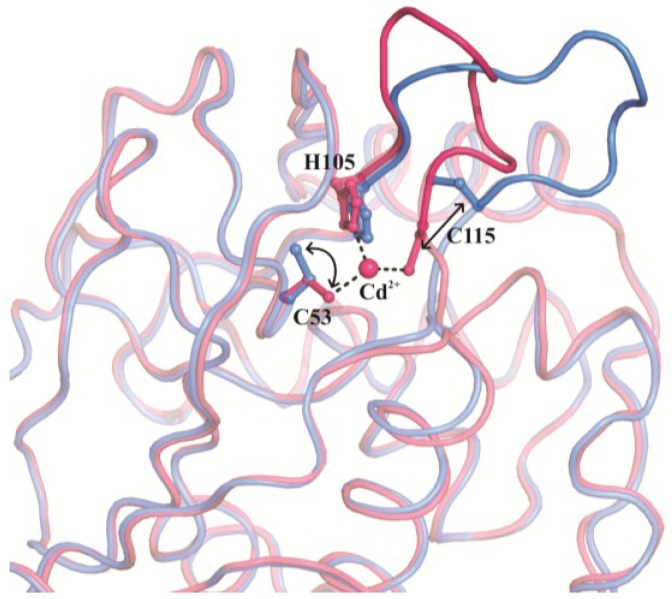
Structural comparison between the Cd-bound (magenta) and metal-free (blue) forms of CDCA1-R1. Cd^2+^ coordinating residues are represented in ball-and-sticks and their displacements are indicated by arrows. The region encompassing the 107–115 sequence is also highlighted.

The crystallographic studies available on CDCA1 allowed us to gain insights into the structural basis for the “facile metal exchange”, which is one of the main features of this cambialistic enzyme. In more detail, the comparison between the structures of the metal-bound and metal-free forms of CDCA1-R1 reveals significant differences in the active site where a conformational change—involving mainly the two metal-coordinating cysteine residues and the region 107–115—takes place, leading in the metal-free form to a more open conformation (Figure 3) [3]. Thus, CDCA1 possesses a stable metal-free form of the active site, which, differently from the other known CAs, is structurally distinct from the metal-bound one.

**Figure 4:**
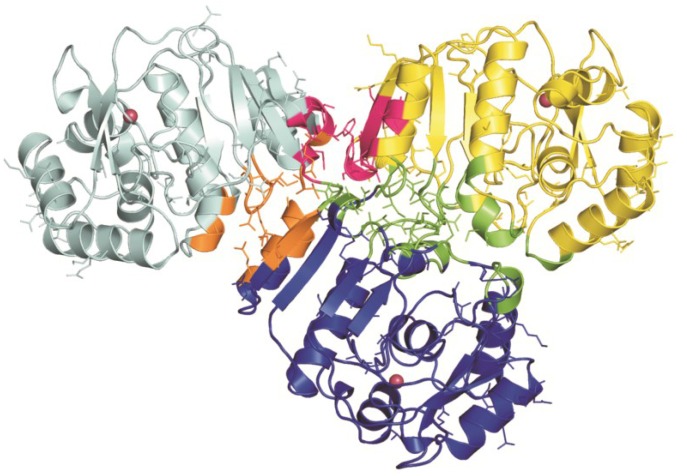
Model of the CDCA1 full length. R1 (cyan), R2 (blue) and R3 (yellow) are shown in cartoon representation. Interface regions are displayed in orange (R1-R2), green (R2-R3) and magenta (R1-R3). Non conserved residues are displayed in ball-and-stick and the three metal ions are reported as pink spheres.

Any attempt to crystallize the full-length CDCA1 protein was unsuccessful, thus, a model was recently proposed by our group [24]. This model, obtained by using a docking approach, shows an asymmetric, compact structure characterized by two covalently linked interfaces (R1-R2 and R2-R3) and a small non-covalent interface (R1-R3) (Figure 4). It is worth noticing that the three active sites are far from each other and completely accessible to the substrate, consistent with Morel’s studies which showed that the catalytic activity of the Cd-full length protein is about three times that of the Cd-CDCA1-R2 repeat [3]. Interestingly, non-conserved residues are mainly located at the interfaces between the three repeats suggesting that they can play a role in stabilizing the global structure of the full-length protein.

## 4. Potential Biotechnological Applications of CDCA1 Enzyme

Heavy metals are ubiquitous elements essential for life, many of them having important roles in the metabolism of living organisms (*i.e.*, zinc, cobalt, copper, and iron for humans) [26,27,28,29]. However, elevated concentrations of both essential and non-essential heavy metals can have a negative influence on the environment and, consequently, can be dangerous for human health [26,30,31,32]. Although the negative effects of heavy metals on the ecosystem have been known for a long time, their production and emission continues and is even increasing in some countries as a consequence of human induced activities, becoming a global concern for their impact on human life [31]. Therefore, many high sensitive, selective, reliable, and accurate analytical methods for the detection and monitoring of metal ions at low concentrations in complex media have been developed and are currently commercially available [33,34]. However, these methods, including atomic absorption and emission spectroscopy, inductively coupled plasma mass spectrometry, anodic and cathodic stripping voltammetry, and their combination with chromatographic techniques, require sophisticated instrumentation ill-suited for use outside the laboratory, skilled personnel, long measuring periods with complicated sample collection and processing [33,34]. An interesting alternative to the classical analytical methods is represented by the recent development of sensors based on biological molecules, termed biosensors. These sensors are characterized by a high affinity for the target metal ion, as well as a high discrimination capability among different transition metals, and also a kinetically rapid biosensor-analyte association and dissociation [33,34,35,36,37,38,39]. Furthermore, biosensor-based measurements have the great advantage to be simple, rapid and inexpensive, permitting a continuous readout of metal ion concentration *in situ* and in real time, since they do not need any processing or separation step [34,37]. In this context, a great interest has been recently shown for carbonic anhydrases (generally human isoform II) for the design of fluorescence based biosensors for the determination of free metals in solution [34,40,41]. From a physical point of view, CA-based biosensors take advantage of the high affinity of the apo-CA for metal ions, in particular for Zn^2+^ [42]. Indeed, the binding of the metal ion to the apo-CA (devoid of any catalytic activity) reconstitutes the catalytically active holo-CA, which can bind a fluorophore inhibitor, leading to a measurable change in the intensity and wavelength of the inhibitor fluorescence emission [43,44]. However, the high affinity of hCA II for Zn^2+^ (4 pM) [42] and the relative abundance of this element in the environment, limits the use of CA-based biosensors for the detection of trace amounts of other metal ions [45]. To circumvent these limitations and to improve the sensitivity, selectivity and affinity of CA-based biosensors for various heavy metals such as Cu^2+^, Co^2+^, Cd^2+^, and Ni^2+^, hCA II variants obtained by site-directed mutagenesis [46,47,48,49] have been produced [41,50,51,52,53].

The enzyme here described, CDCA1, which has a natural high affinity for Cd^2+^ and a good capability to bind sulfonamide inhibitors, could represent an interesting alternative to CA II-based biosensors for the detection of Cd^2+^ trace amounts in marine environment [3]. As for hCA II, CDCA1 variants with a lowered binding affinity for Zn^2+^ need to be developed by structure-based design, before detection of cadmium ions can be feasible. Even though this is a difficult task, examples of single site mutagenesis on CDCA1-R2 residues (G316A and G324A) flanking the linkage sequence between two metal binding residues proved to be effective in modulating Zn/Cd metal affinity, resulting in a loss of ability to exchange the two metals [3]. In addition, the design and synthesis of ligands containing highly affine and selective Cd-binding groups, *i.e.*, exploiting the considerably more thiophilic property of cadmium compared to zinc, could help to discriminate between Cd- and Zn-forms of CDCA1 [54].

The very high catalytic activity of CDCA1 for the CO_2_ hydration reaction [3] makes this enzyme also a useful tool for the design of bioreactor systems for carbon dioxide capture and its conversion into water soluble ions. This biomimetic method requires robust CAs that can work at high pH and temperature, being minimally inhibited by other gases and ions. Thus, thanks to its high catalytic efficiency at high pH values and ability to properly work in seawater where high concentrations of marine ions are present [3], CDCA1 could be a good candidate for the development of biotechnological tools for the environmental defense in the current global warming scenario linked to elevated CO_2_ production.

## 5. Conclusions

In this review, we have presented the biochemical and structural features of CDCA1 enzyme which has been identified in *Thasassiosira weissflogii*. This enzyme, being completely different from any other CA, represents the first member of a new class, the ζ-class. CDCA1 enzyme naturally uses Cd^2+^ to achieve its biological function, providing the first evidence of the biological employment of this metal ion, usually considered a toxic element associated to environment contamination. The high affinity of CDCA1 for Cd^2+^, as well as its high catalytic activity for the physiological reaction, makes this enzyme appealing for the development of biotechnological tools to monitor water and air pollution.
